# Validation of a quantitative lateral flow immunoassay (LFIA)-based point-of-care (POC) rapid test for SARS-CoV-2 neutralizing antibodies

**DOI:** 10.1007/s00705-022-05422-w

**Published:** 2022-04-02

**Authors:** Massimo Pieri, Eleonora Nicolai, Marzia Nuccetelli, Serena Sarubbi, Flaminia Tomassetti, Martina Pelagalli, Marilena Minieri, Alessandro Terrinoni, Sergio Bernardini

**Affiliations:** 1 Department of Experimental Medicine, University of Rome “Tor Vergata”, Via Montpellier 1, 00133 Rome, Italy; 2 Department of Laboratory Medicine, “Tor Vergata” University Hospital, Viale Oxford 81, 00133 Rome, Italy

## Abstract

With the widespread use of coronavirus disease 2019 (COVID-19) vaccines, a rapid and reliable method to detect SARS-CoV-2 neutralizing antibodies (NAbs) is extremely important for monitoring vaccine effectiveness and immunity in the population. The purpose of this study was to evaluate the performance of the RapiRead™ reader and the TestNOW™ COVID-19 NAb rapid point-of-care (POC) test for quantitative measurement of antibodies against the spike protein receptor-binding domain of severe respiratory syndrome coronavirus 2 (SARS-CoV-2) in different biological matrices compared to chemiluminescence immunoassay (CLIA) methods. Ninety-four samples were collected and analyzed using a RapiRead™ reader and TestNOW™ COVID-19 NAb kits for detecting neutralizing antibodies, and then using two CLIAs. The data were compared statistically using the Kruskal-Wallis test for more than two groups or the Mann-Whitney test for two groups. Specificity and sensitivity were evaluated using a receiver operating characteristic (ROC) curve. Good correlation was observed between the rapid lateral flow immunoassay (LFIA) test system and both CLIA methods. RapiRead™ reader/TestNOW™ COVID-19 NAb vs. Maglumi: correlation coefficient (r) = 0.728 for all patients; r = 0.841 for vaccinated patients. RapiRead™ reader/TestNOW™ COVID-19 NAb vs. Mindray: r = 0.6394 in all patients; r = 0.8724 in vaccinated patients. The time stability of the POC serological test was also assessed considering two times of reading, 12 and 14 minutes. The data revealed no significant differences. The use of a RapiRead™ reader and TestNOW™ COVID-19 NAb assay is a quantitative, rapid, and valid method for detecting SARS-CoV-2 neutralizing antibodies and could be a useful tool for screening studies of SARS-CoV-2 infection and assessing the efficacy of vaccines in a non-laboratory context.

## Introduction

Coronavirus disease 2019 (COVID-19) represents the largest public health emergency in the last two years. With the spread of COVID-19 vaccines, it has become of central importance for laboratories to assess immunity and protection against severe acute respiratory syndrome coronavirus 2 (SARS-CoV-2), and the use of antibody testing is an essential tool in the vaccination campaign to promote public health [1]. So far, data on the duration of immunity generated by SARS-CoV-2 infection or vaccination are still limited. More information on the response to vaccination could help to evaluate its efficacy and to determine whether booster shots are needed. A large number of different methods and technical approaches have been devised to measure the immune response and antibody kinetics to SARS-CoV-2 infection [2–4]. To evaluate the efficacy of COVID-19 vaccines and to monitor the level of protective neutralizing antibodies, it is necessary to develop a diagnostic tool that is easy to use and at the same time is accurate and provides useful information about the duration of immunity.

Lateral flow immunoassay (LFIA)-based point-of-care (POC) serological tests have been developed to detect anti-SARS-CoV-2 antibodies. In contrast to chemiluminescent serum tests, POC serological tests do not require technical personnel or laboratory equipment, are inexpensive, and provide results quickly. Furthermore, the risks associated with sampling and specimen preparation are greatly reduced while retaining high sensitivity and high specificity [5, 6].

Commercially available enzyme immunoassays can detect neutralizing antibodies with high diagnostic accuracy, whereas measuring neutralizing antibodies *in vitro* requires highly laborious assays performed in biosafety facilities and is limited to research institutions [7, 8].

POC testing can be performed in a variety of settings, including physicians’ offices, emergency rooms, urgent care facilities, school clinics, and pharmacies [9].

However, the benefit of these rapid serological POC LFIA-based tests has not been widely studied. Among the anti-SARS-CoV-2 antibodies detected in binding assays, neutralizing antibodies (NAbs) that block the interaction between SARS-CoV-2 and its human receptor ACE2 (angiotensin-converting enzyme 2) are of particular importance with regard to vaccination. A robust and rapid method for detection of SARS-CoV-2 neutralizing antibodies can be widely employed to investigate SARS-CoV-2 infection and assess the efficacy of vaccines.

The RapiRead reader is currently the world’s smallest LFIA reader system for measuring spike protein receptor binding domain (S-RBD) antibody levels, and it shows good correlation with the World Health Organization International Standard (WHO-IS) for anti-SARS-CoV-2, with results given in binding antibody units (BAU).

The aim of this study was to evaluate the performance of the RapiRead™ reader and the TestNOW™ COVID-19 NAb test, using different biological matrices, and to compare the data obtained using these assays with those obtained using chemiluminescence immunoassay (CLIA) methods for SARS-CoV-2 S-RBD antibody detection.

## Materials and methods

### Patients

Ninety-four samples were analyzed in this study. Serum samples were collected from 25 patients with SARS-CoV-2 infection confirmed by RT-PCR and 39 vaccinated health workers from Tor Vergata University Hospital of Rome who had received the second inoculation of the Pfizer vaccine at least 21 days earlier. For each individual, peripheral blood, EDTA plasma, and serum samples were collected; 30 presumed-negative serum samples collected before the SARS-CoV-2 outbreak, stored at – 80 °C were used as a negative control.

The study was conducted in accordance with the guidelines of the local ethics committee (approval number: R.S.44.20) and the Helsinki Declaration, as revised in 2013.

### TestNOW^®^- COVID-19 NAb

TestNOW^®^- COVID-19 NAb (Affimedix Inc., CA, USA) uses the principle of immunochromatography for detection of NAbs. When the sample migrates through the membrane, the conjugate, consisting of colored RBD (the target) and colloidal gold, forms a complex with specific NAbs against SARS-CoV-2, if present in the sample. This complex migrates further along the membrane to the “T” (test) zone, where mouse anti-human IgG antibodies are immobilized on the nitrocellulose membrane on the cassette. There, the conjugate is “captured” by anti-human IgG antibodies bonded to the membrane, leading to the formation of a colored band, indicating a positive test result. The intensity of the colored band in the test line area depends on the concentration of NAbs present in the sample. A built-in control line (C) will always appear in the test window when the test has performed properly, regardless of the presence or absence of NAbs against SARS-CoV-2 in the specimen.

### RapiRead™ reader

The RapiRead reader (Affimedix Inc., CA, USA) is utilized for reading the intensity of the colored band. For quantitative diagnostics, the intensities of the test lines are compared to a calibration standard and converted to an analyte concentration value. The instrument measures reflective optical density by taking multiple images through an LED (light-emitting diode) camera and recording reflectance of the test strip surface. The reader uploads the calibration file wirelessly using an RFID (radio frequency identification) card and can operate in stand-alone mode without a computer or external power source. The cutoff of TestNOW^**®**^ is ~ 30 BAU/mL, depending on the specific limit of detection (LOD) of the particular production lot. According to the manufacturer, values < 30 BAU/mL are considered negative; values of 30-250 BAU/mL indicate low protection; values of 250-500 BAU/mL indicate medium protection; and values > 500 BAU/mL indicate high protection.

### Mindray SARS-CoV-2 S-RBD IgG

Mindray SARS-CoV-2 S-RBD IgG (Mindray S-RBD IgG) is a two-step CLIA for quantitative determination of SARS-CoV-2 S-RBD IgG in human serum or plasma, performed on the fully automated Mindray CL 1200i analytical system (Mindray Bio-Medical Electronic Co Ltd, Shenzhen, China). According to the manufacturer, the cutoff value is 12.16 BAU/mL, and the linear range is 3.65-1216 BAU/mL. Samples with values over 1216 BAU/mL were diluted 1:10 before measurement, allowing extension of the dynamic range of analysis to 12,160 BAU/mL.

### Maglumi SARS-CoV-2 S-RBD IgG

Maglumi SARS-CoV-2 S-RBD IgG (Snibe S-RBD IgG) is an indirect CLIA for *in vitro* quantitative determination of IgG antibodies to SARS-CoV-2 S-RBD, performed using a fully automated Maglumi 800 analytical system (Snibe Diagnostic, Shenzhen, China). According to the manufacturer, the cutoff value is 4.33 BAU/mL, and the linear range is 0.78-433 BAU/mL. Samples with values over 433 BAU/mL were diluted and measured at 1:10 or 1:20 (if necessary), allowing extension of the dynamic range of analysis to 8660 BAU/mL.

### Statistical analysis

Descriptive analyses were performed, with measures of central tendency and dispersion for continuous variables and frequency distribution for qualitative variables. In the case of normally distributed data, they were represented by the mean ± standard deviation, and ANOVA with the Bonferroni *post hoc* test was used to determine the significance of differences between more groups. Otherwise, if only two groups were present, Student’s *t*-test was used. In the case of non-normally distributed data, the data were represented as the median and the percentiles. The variables were compared using the Kruskal-Wallis test for more than two groups or the Mann-Whitney test for two groups. Shapiro-Wilk test was used for testing the normality of data with a 95% confidence interval (CI).

Receiver operating characteristic (ROC) curve analysis was used to determine the specificity and sensitivity relative to the cutoff suggested by the manufacturer.

All data were examined using Med Calc Ver.18.2.18 (MedCalc Software Ltd, Ostend, Belgium).

The statistical significance level established for all tests performed was *p* < 0.05.

## Results

In our study, RapiRead™ reader/TestNOW™ COVID-19 NAb data were compared with those obtained by two CLIA methods for SARS-CoV-2 S-RBD antibody detection using different biological matrices (peripheral blood, EDTA plasma samples, and serum samples).

**Fig. 1:**
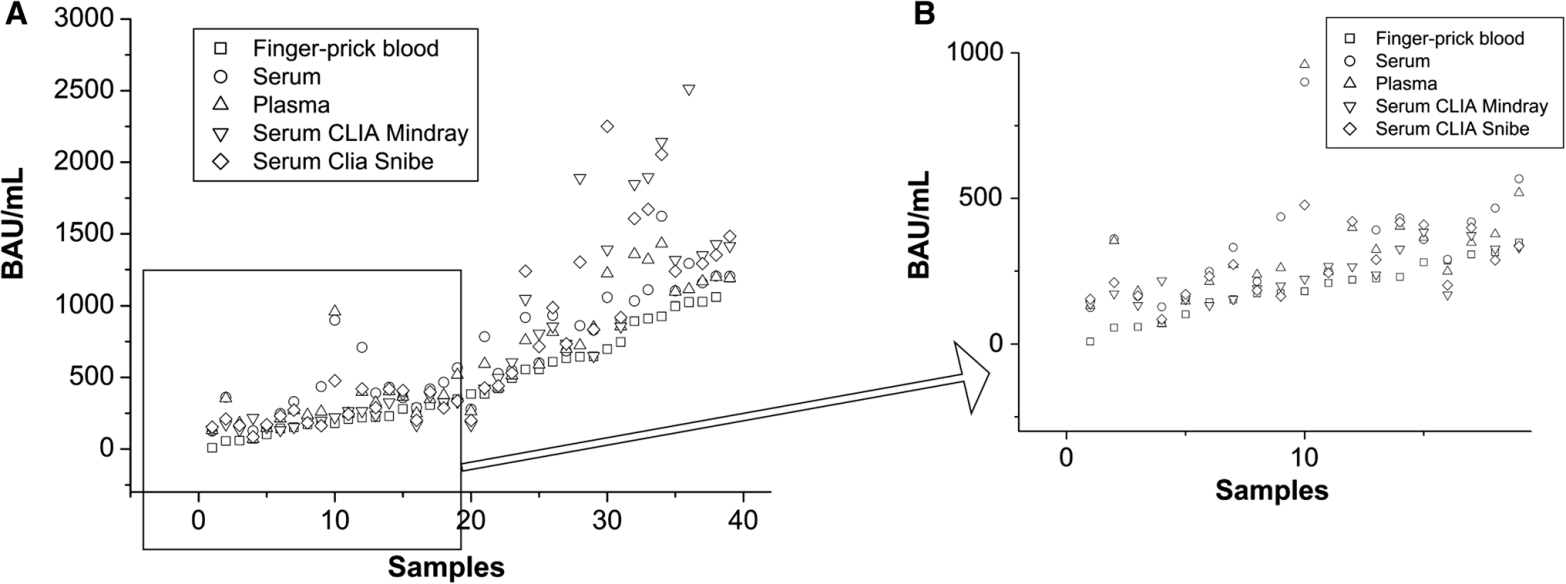
(**A**) Scatter plot graph of all data: finger-prick blood, serum matrix, plasma matrix, serum CLIA Mindray, serum CLIA Snibe. (**B**) An enlargement of the figure showing details for samples from 1 to 20

The scatter plot in Figure 1A shows good correlation between the Affimedix rapid test and both CLIA methods, Snibe and Mindray. Different biological matrices were compared, and finger-prick blood, serum, and plasma samples showed no significant difference. Data were sorted from the lowest to the highest value, from 0 to 2500 BAU/mL. Figure 1B shows an enlargement of the first 20 samples, showing a good alignment up to about 500 BAU/mL.

Since good correlation was observed in the overall set of samples, it was evaluated whether linearity was maintained in the serum matrix between the RapiRead™/TestNOW™ system and the other two platforms, evaluated individually.

**Fig. 2:**
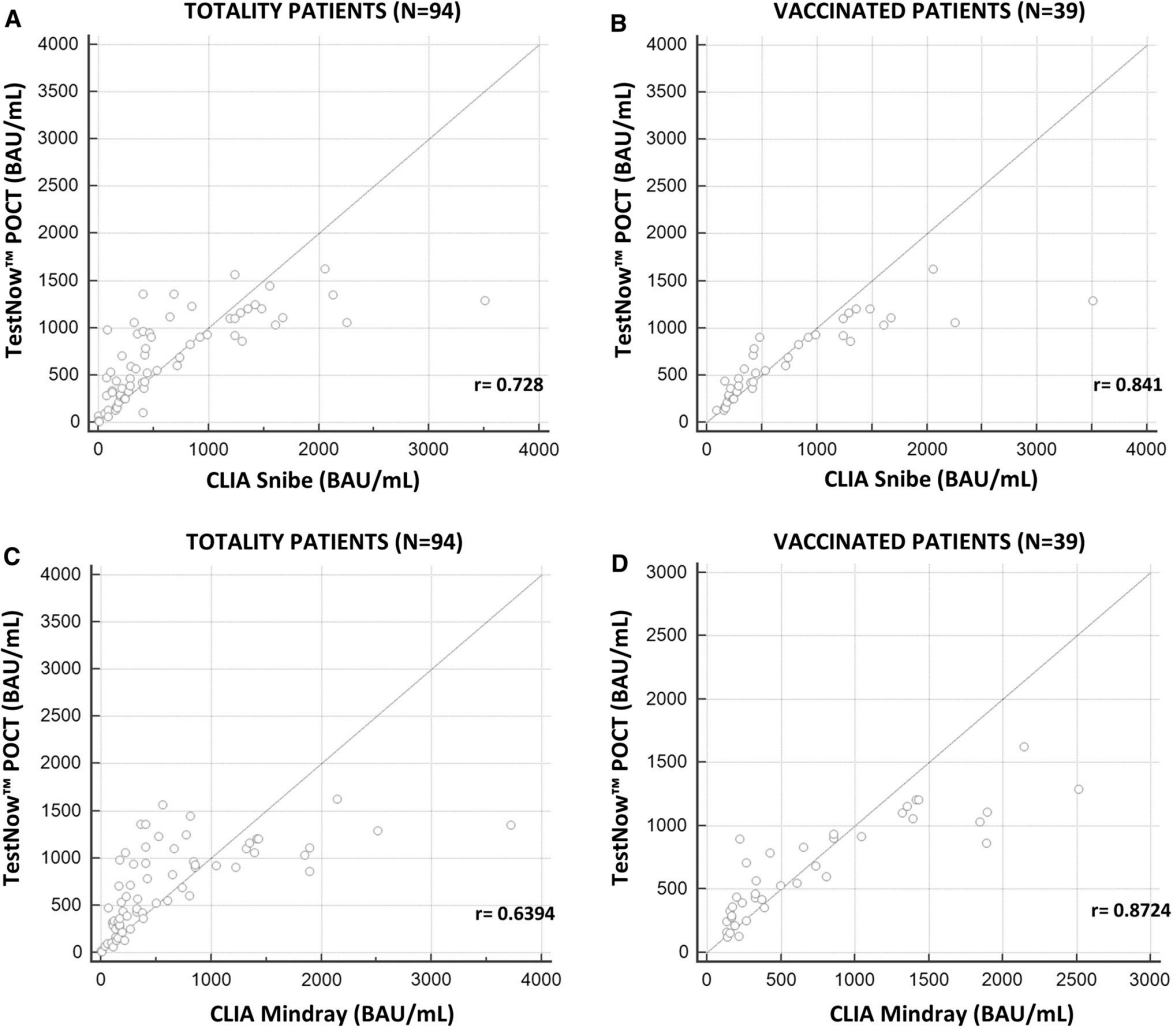
(**A**) Linear regression between TestNow™ POCT and Snibe Maglumi for all patients. (**B**) Linear regression between RapiRead™ reader/TestNow™ and Snibe Maglumi for vaccinated patients. (**C**) Linear regression between RapiRead™ reader/TestNow™ and Mindray 1200i for all patients. (**D**) Linear regression between RapiRead™ reader/TestNow™ and Mindray 1200i for vaccinated patients

Figure 2 shows that there was a strong correlation between the RapiRead™ reader/TestNow™ and CLIA methods when testing serum samples. The data were evaluated separately for all of the patients (n = 94) and just the vaccinated patients (n = 39). A significant correlation was observed between RapiRead™ reader/TestNow™ and Snibe Maglumi, with a correlation coefficient (r) of 0.728 for all of the patients and 0.841 for the vaccinated patients (Fig. 2A and B).

Also, the relationship between TestNow™ and Mindray 1200i showed a significant correlation, with an r-value of 0.6394 for all of the patients and 0.8724 for the vaccinated patients (Fig. 2C and D).

**Fig. 3:**
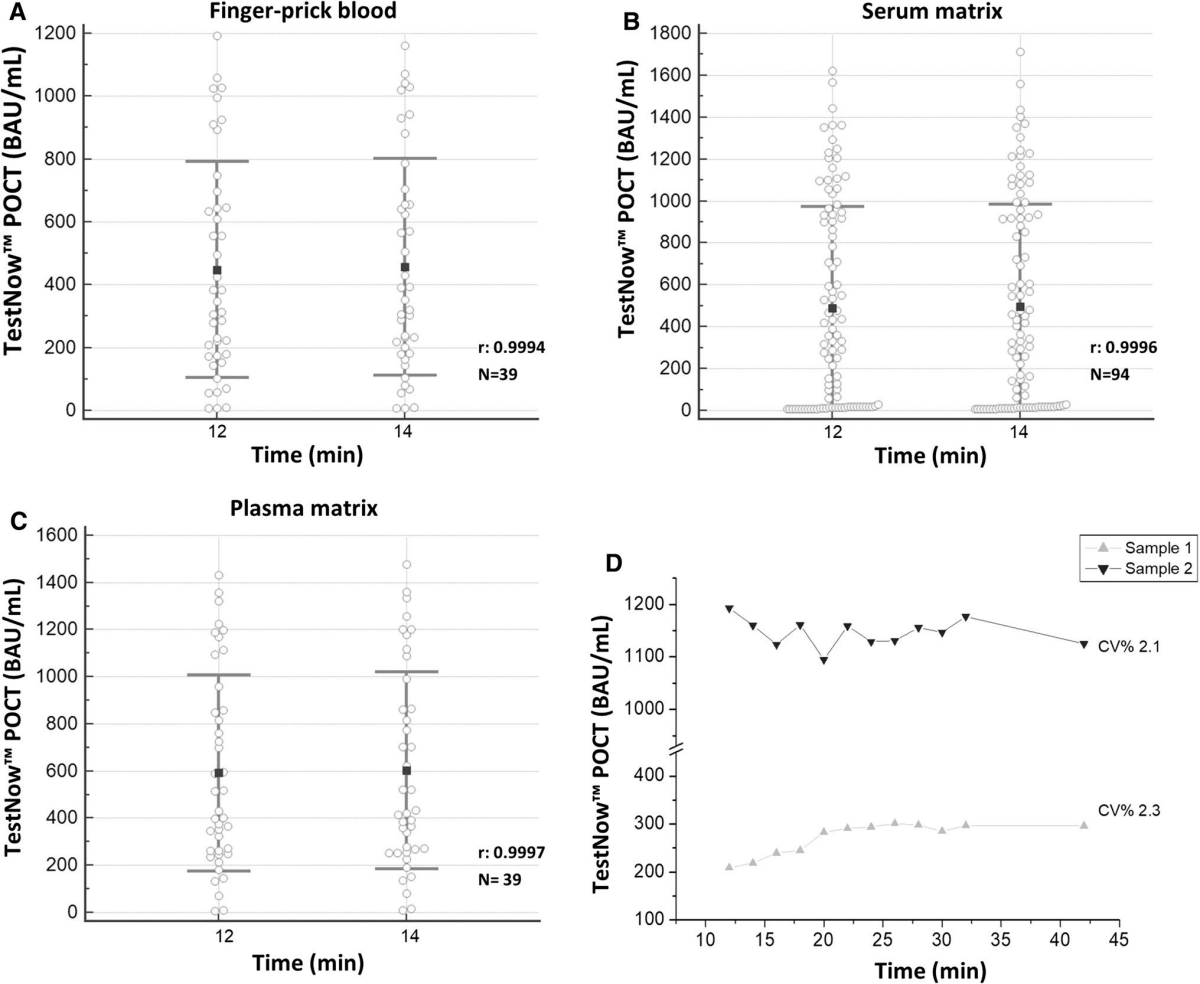
(**A**) Statistical data for reader measurements at two different time points, using finger-prick blood: mean at 12 min = 470.55 BAU/mL; mean at 14 min = 497.04 BAU/mL; r = 0.9994 (*p* < 0.0001). (**B**) Statistical data for reader measurements at two different time points, using serum matrix: mean at 12 min = 497.21 BAU/mL; mean at 14 min = 506.09 BAU/mL; r = 0.9996 (*p* < 0.0001). (**C**) Statistical data for reader measurements at two different time points, using plasma matrix: mean at 12 min = 621.87 BAU/mL; mean at 14 min = 632.63 BAU/mL; r = 0.9997 (*p* < 0.0001). (**D**) A dot-plot graph with time linearity up to 42 min between two samples

Furthermore, the time stability of the POC serological test reader procedure was assessed. The manufacturer recommends reading the test results within 15 min. In this study, we considered two times of reading: 12 and 14 min. As shown in Figure 3, the correlation between two different reading times (12 and 14 min) with different matrices from the same individual (finger-prick blood, serum, and plasma) showed no significant differences, with correlation coefficients of 0.9994, 0.9996, and 0.9997 (*p* < 0.0001), respectively (Fig. 3A-C).

Figure 3D illustrates two samples with readings taken at time points after 14 min. The two samples with readings up to 42 min have coefficients of variation of 2.1% and 2.3% respectively, confirming the stability of the reading beyond the times recommended by the manufacturer.

**Fig. 4:**
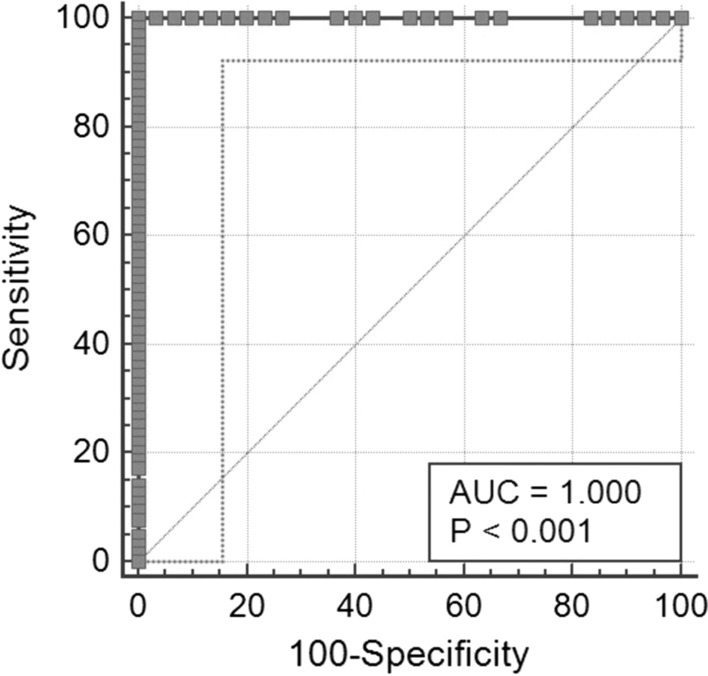
RapiRead™ reader/TestNow™ ROC curve. 95% confidence interval (CI): 0.962 to 1.00

Lastly, Figure 4 shows a receiver operating characteristic (ROC) analysis to assess specificity and sensitivity with respect to the cutoff suggested by the manufacturer. Data obtained using serum samples were evaluated: 30 control samples from RT-qPCR-diagnosed SARS-CoV-2-negative and unvaccinated patients; 25 from SARS-CoV-2-positive patients, from RT-qPCR-diagnosed SARS-CoV-2 patients who were positive for more than 30 days, and 39 vaccinated patients.

The ROC curve showed that the sensitivity and specificity were both 100%, with an area under the curve (AUC) value of 1.

## Discussion

With the spread of SARS-CoV-2, rapid serological tests have been largely applied for detection and quantitation of antibodies [7]. Hundreds of point-of-care tests (POCTs) have been developed and are commercially available. Among the immunoassays, LFIAs are the fastest and most convenient tests, usually requiring only 15 min to complete. They can be performed by a professional, either in a laboratory or at a remote site, and can therefore complement existing NAb tests. In this study, we evaluated TestNow™ COVID-19 NAb to assess its power of detection and performance. The tool proved to be the quickest and easiest way to detect neutralizing antibodies that block the interaction between SARS-CoV-2 virus and ACE2, with a good correlation with the chemiluminescence tests, using the different sample types from the same individuals: finger-prick blood, serum, and plasma.

Correlation coefficients of 0.7280 and 0.6394 were obtained with Snibe Maglumi and Mindray 1200i, respectively. When considering samples only from vaccinated individuals (n = 39; serum samples), the correlation coefficients, were 0.841 and 0.872 with Snibe Maglumi and Mindray 1200i, respectively. The strong linearity obtained with samples from vaccinated patients was due to the exclusion of the highest values from infected patients, which were not detectable by RapiRead™ reader/TestNow™ POCT.

An additional value of this new device is a direct readout in BAU/mL, the international standard, following NIBSC (National Institute for Biological Standards and Control, UK) [10] or WHO-IS guidelines [11], and this allows an immediate comparison with other methods.

According to the manufacturer, measurements should be taken after an incubation time of 15 min, but we observed that acceptable results could be achieved over a longer range of times. The data did not show any significant difference between readings taken at 12 or 14 min. This wider range would improve the tool performance, guarantee the stability of results, and simplify the workflow.

Finally, ROC analysis confirmed the manufacturer’s cutoff, with optimal sensitivity and specificity of 100%. Unfortunately, this study was limited by a small sample size, and the cutoff values should be confirmed with a larger cohort of patients in future studies.

The results demonstrate that TestNow™ COVID-19 NAb is a quantitative, valid, rapid, and simple method to detect SARS-CoV-2 NAb levels that could be employed widely for screening studies of SARS-CoV-2 infection assessing the efficacy of vaccines. The ability to insert the lateral flow cartridge into the instrument and obtain a quantitative readout can be used to complement its use as a stand-alone assay for measuring antibody levels in non-laboratory settings such as workplaces, hospitals, residential facilities, schools, or other locations where a rapid result is needed.
